# LincRNA-EPS in biomimetic vesicles targeting cerebral infarction promotes inflammatory resolution and neurogenesis

**DOI:** 10.1186/s12967-020-02278-z

**Published:** 2020-03-02

**Authors:** Benping Zhang, Qian Li, Shuwei Jia, Feng Li, Qingsong Li, Jiebing Li

**Affiliations:** 1 Department of Neurology, The Second Affiliated Hospital, Harbin Medical University, Harbin, 150086 Heilongjiang People’s Republic of China; 2 Department of Biopharmaceutical Sciences, College of Pharmacy, Harbin Medical University, Harbin, 150086 Heilongjiang People’s Republic of China; 3 Department of Physiology, Harbin Medical University, Harbin, 150086 Heilongjiang People’s Republic of China; 4 Departments of Neurosurgery, The Second Affiliated Hospital, Harbin Medical University, Harbin, 150086 Heilongjiang People’s Republic of China; 5 Department of Ultrasound, Harbin Medical University Cancer Hospital, Harbin, 150086 Heilongjiang People’s Republic of China

**Keywords:** Biomimetic proteolipid vesicles, Inflammation, LincRNA-EPS, Neuron regeneration, Stroke

## Abstract

**Background:**

Inflammatory damage following stroke aggravates brain damage, resulting in long-term neurological sequelae. The purpose of this study was to identify ways to reduce inflammatory reactions and to accelerate neuron regeneration after cerebral apoplexy.

**Methods:**

We formulated a biomimetic vesicle, the leukosome, constituted by liposome, artificial long intergenic noncoding RNA (lincRNA)-EPS, and membrane proteins derived from macrophages and their physical–chemical characteristics were evaluated. Migration distance and cytotoxic levels were measured to determine the effect of lncEPS-leukosomes on lipopolysaccharide-activated microglia. An in vivo transient middle cerebral artery occlusion/reperfusion (tMCAO) model was established in mice, which were treated with lncEPS-leukosomes. Vesicle seepage, infiltration of inflammatory cells, cytotoxic levels in the cerebrospinal fluid, and neural stem cell (NSC) density were measured.

**Results:**

Biomimetic vesicles with a homogeneous size increased lincRNA-EPS levels in activated microglia by 77.9%. In vitro studies showed that lincRNA-EPS inhibited the migration and cytotoxic levels of activated microglia by 63.2% and 43.6%, respectively, which promoted NSC proliferation and anti-apoptotic ability. In vivo data showed that leukosomes targeted to inflamed sites and lncEPS-leukosomes decreased the infiltration of inflammatory cells and cytotoxic levels by 81.3% and 48.7%, respectively. In addition, lncEPS-leukosomes improved neuron density in the ischemic core and boundary zone after tMCAO.

**Conclusions:**

The biomimetic vesicles formulated in this study targeted inflammatory cells and accelerated neuron regeneration by promoting inflammation resolution. This study may provide a promising treatment approach for accelerated neuron regeneration after cerebral apoplexy.

## Background

Stroke poses a significant threat to human health because of its high rate of incidence and severe disability [1–5]. After cerebral ischemia, activated microglia release large amounts of inflammatory cytokines and cytotoxic factors, which not only cause neuronal damage, but also inhibit the maintenance of neural stem cells (NSC), and promote the conversion to astrocytes [6–9]. The barrier function of the blood brain barrier (BBB) combined with the sensitivity and vulnerability of cerebral balance [10], makes finding a safe and effective therapeutic regimen challenging. Increasing the dose of neuron-protective medicines may increase availability to the target tissue; however, heavy-duty medicines significantly increase the risk of diabetic retinopathy, rheumatoid arthritis, and neoplasm [11]. Therefore, targeting adequate delivery of neuron-protective medicines to the cerebral ischemia zone has become a major focus of current research.

Recently, liposomes have increasingly been used as a drug delivery system. Liposomes can not only cross the BBB due to its oleophilic and hydrophilic structure, they also enhance utilization of the targeting drugs and efficiently improve drug stability [12–17]. However, as an exogenous vesicle, liposomes may be ingested by peripheral phagocytes, which limit effective and targeted treatment. Thus, creating liposomes that evade immune system surveillance and enhance targeting treatment is a major challenge [18–20].

At present, many traditional protective medicines for the treatment of stroke have a central nerve-depressant effect, and may produce several side-effects while protecting nerves. Long intergenic noncoding RNA (lincRNA) has many gene regulatory effects that were previously unrecognized. For example, in macrophages, lncRNA-EPS is precisely regulated to control the expression of immune response genes (IRGs). In previous studies, it was demonstrated that lincRNA-EPS suppressed inflammation by inhibiting gene expression of components of the immune response. Macrophages and dendritic cells express lincRNA-EPS, however, this is downregulated in cells stimulated with microbial ligands, including lipopolysaccharide (LPS) [21–23]. In addition, lincRNA-EPS specifically acts on the immune system, suggesting that lincRNA-EPS may be an ideal anti-inflammatory molecule [24].

In this study, we hypothesized that treatment with lincRNA-EPS is a promising strategy to inhibit inflammation and promote neuron regeneration. Previous studies have shown that several adhesion molecules presented on the cell surface of leukocytes help nanoparticles evade immune system surveillance. In this study, we extracted membrane proteins from macrophages (Mφ) [25–28], and constructed so-called ‘leukosomes’ that were loaded with lincRNA-EPS, a type of manufactured Mφ, by phosphocholine-based phospholipids (DPPC, DSPC and DOPC), cholesterol (Avanti Polar Lipids), and the above-mentioned proteins.

Injection of lnc-EPS-leukosomes into the body, caused homing of the lnc-EPS-leukosomes to the inflammatory zone of brain tissue, resulting in the release of lincRNA-EPS to regulate inflammation and promote neuron regeneration. In our study, we developed a novel targeting carrier for microglia with an lnc-EPS-loaded liposome. Our findings suggested that lnc-EPS-leukosomes may be a promising, effective therapy for the treatment of stroke.

## Materials and methods

### Membrane proteins of Mφ incorporate with lipid vesicles

All animal experiments were reviewed and approved by the Institutional Review Board Service and Institutional Clinical Experiments Committee of Harbin Medical University (Heilongjiang, China). In addition, all methods were performed in accordance with relevant guidelines and regulations. Twenty 10–14-week-old male C57BL/6 mice were obtained from the Experimental Animal Center of the Harbin Medical University (Heilongjiang, China) and were sacrificed by CO_2_ inhalation. Sterile phosphate buffer saline (PBS) was injected into the abdominal cavity, the belly was gently massaged, and the liquid aspirated. Cells were collected and incubated in red blood cell lysis buffer for 2 min, after which membrane proteins of Mφ were extracted by ProteoExtract Native Membrane Protein Extraction Kit (Merck, Darmstadt, Germany) and dispersed in PBS [29]. Liposomes and leukosomes were incubated with mouse-anti-CD68 (BD Biosciences, New York, New Jersey, USA) at 4 °C for 12 h, followed by washing with PBS, and incubation with a Rhoda-mouse-IgG secondary antibody at room temperature for 15 min (ThermoFisher, Waltham, Massa Chusetts).

Lnc-EPS-leukosomes were produced using the method of thin layer evaporation [30, 31]. The proportion used was as follows: 5:3:1:1, which represented 1,2-dipalmitoyl-sn-glycero-3-phosphocholine (DPPC, Resenbio, Xi’an, China); 1,2-dioleoyl-sn-glycero-3-phosphocholine (DOPC, Resenbio, Xi’an, China); 1,2-distearoyl-sn-glycero-3-phosphocholine (DSPC, Resenbio, Xi’an, China) and cholesterol (Avanti Polar Lipids, Alabaster, AL, USA). The above-mentioned compounds were dissolved in chloroform. Using a rotary evaporator (BÜCHI Labortechnik AG, Switzerland), the solvent was evaporated to form a thin film. The film was hydrated with a protein solution and lincRNA-EPS (Genebio, Shanghai, China) to generate lnc-EPS-leukosomes by revolution at 45 °C for 3 min. The membrane proteins to lincRNA-EPS to lipid ratio was 1:3:300. A liposome extruder (LiposoEasy LE-1, Morgec Machinery, Shanghai, China) was used to prepare vesicles that were uniform in size. Obtained vesicles were dialyzed for 12 h to eliminate unwanted proteins, lincRNA-EPS, and lipids through 1000KDa membranes (Spectrum Laboratories lnc, Los Angeles, California, USA). Lnc-EPS-leukosomes were stored at 4 °C.

### Dynamic light scattering and cryo-electron microscopy analysis

Dynamic light scattering analysis was used to determine the polydispersity index, zeta potential, and vesicle size using nanoparticle tracking analysis (NTA, Nano series, Malvern, UK). Measurements were performed in 10 runs per each construct after 20 μl of liposomes or leukosomes were diluted in deionized water.

For cryo-electron microscopy (cryo-EM) analysis, liposomes or leukosomes were rapidly frozen in liquid nitrogen. Next, a R2×2 Quantifoil electron microscope (Micro Tools GmbH, Jena, Germany) was used to collect morphological information at a nominal magnification of 20,000×, and a charged coupled device (CCD) camera (PXL CCD camera, Photometrics, Tucson, AZ) was used for imaging purposes at low electron-dose conditions (~ 5–20 electrons/Å2) [32].

### Isolation and culture of cells

Two days after birth, 40 C57BL/6 mice were sacrificed by overdose of 2.5% sodium pentobarbital (36 mg/kg, Sigma, St. Louis, MO, USA). The brains were isolated, washed with PBS, and then meninges were stripped carefully. The bilateral cerebral cortex was digested for 15–30 min in trypsin, cells were harvested, and filtered by a cyto-screener. Cells were seeded at a density of 5 × 10^5^/ml in 6-well plates (Corning, New York, NY, USA). DMEM/F12 medium (Invitrogen, Carlsbad, CA, USA), containing 10% FCS (Invitrogen, Carlsbad, CA, USA) was used for inoculation and maintenance of cells. After 2 days, 6-well culture plates were placed on a rotatory shaker at 200 rpm for 2 h at 37 °C. Then, cells that were still present in the medium were seeded into other 6-well culture plates. The cells that adhered after 1 h, were the microglia that were used in this study [33, 34].

NSC (CP-M139, C57BL/6, Procell, Wu han, China) were cultured in DMEM/F12 medium, containing EGF (20 ng/ml, Invitrogen, Carlsbad, CA, USA), bFGF (20 ng/ml, Invitrogen, Carlsbad, CA, USA), B-27 (2%, Invitrogen, Carlsbad, CA, USA) and antibiotic (1%, Invitrogen, Carlsbad, CA, USA).

For in vitro experiments, microglia and NSCs were divided into three groups and stimulated with either 1 µg/ml lipopolysaccharide (LPS, Sigma-Aldrich, St. Louis, MO, USA) (*n *= 8); LPS and 40 nM leukosomes (*n *= 8); or LPS for 48 h at 37 °C and 40 nM lnc-EPS-leukosomes for least 24 h (n = 8). The control group was treated with an equal volume of PBS and medium (n = 8).

### Real-time PCR

The GoldScript one-step RT-PCR Kit (Life Technologies, Carlsbad, USA) was used to measure the intracellular lincRNA-EPS content in microglia at 1, 3, 6, 12, 24, 36, and 48 h after the start of culture. In addition, the RNA content of lincRNA-EPS-leukosomes, which absorbed 100 µg lincRNA during the production process, were measured by the RT-PCR kit at 0, 6, 12, 24, and 48 h after production of lincRNA-EPS-leukosomes. Procedures were strictly carried out by following the kit instructions. In addition, the gene expression of Klf4, Oct4, and Sox2 in NSCs were measured by the method mentioned above. As a housekeeping gene, β-action was used. Data were obtained by a Real-Time PCR Instrument (QuantStudio™ 3&5, ThermoFisher, Waltham, MA, USA).

Primers were obtained from Genebio (Shanghai, China), and the primer sequences of Klf4, Oc4, and Sox2 were as follows:

Klf4 5′-GAGCCCAAGCCAAAGAGG-3′/5′-ATCCACAGCCGTCCCAGTC-3′;

Oct4 5′-CAGTGCCCGAAACCCACAC-3′/5′-GGAGACCCAGCAGCCTCAAA-3′;

Sox2 5′-ACACCAATCCCATCCACACT-3′/5′-GCAAACTTCCTGCAAAGCTC-3′.

### Scratch wound assay

A total of 1 × 10^6^ microglia were seeded into 12-well culture plates and cultured overnight, after which mitomycin (5 µg/ml, Bio-Rad Laboratories, Hercules, CA, USA) was added for 2 h. Then a sterile P-200 pipet tip was used to create an artificial wound in each well. Microglia in each group were stimulated as described above. After 12 h of culturing, cells were stained with crystal violet, evaluated by light microscopy (Leica, DVM6, Solms, Germany) and data were analyzed using ImageJ software (National Institutes of Health (NIH, Bethesda, MD, USA).

### Co-culture in vitro

A total of 1 × 10^6^ microglia were harvested and placed into the upper chamber of a 12-well trans-well plate (0.65 µm, Corning, New York, NY, USA), containing 1 µg/ml LPS. In addition, 1 × 10^6^ NSCs were placed into the lower chamber, wells contained either culture medium; 40 nM leukosomes; or 40 nM lnc-EPS-leukosomes (*n *= 8). The microglia and medium in the control group were not untreated.

After culturing for 24 h at 37 °C, NSCs were cultured overnight using EdU medium (Ribobio, Guangzhou, China) and treated with 0.3% Triton X-100 in 1% fetal bovine serum (FBS) for 15 min at 37 °C. Then, cells were washed with PBS and to stain mitotic NSCs, 100 μL 1 × Apollo-567 (Ribobio, Guangzhou, China) was added to each well. DAPI was used to stain nuclei. Cells were evaluated and imaged using a fluorescence microscope (DMI4000B, Leica, Germany) at 565 nm and 461 nm.

The grouping and stimulation methods were identical as mentioned above. After 48 h, NSC were harvested, washed, and resuspended in PBS. A FITC-conjugated Annexin V Apoptosis Detection Kit (BD Biosciences, Franklin, NJ, USA) was used to identify apoptotic NSCs. Procedures were strictly performed by following the kit instructions, and cell apoptosis was evaluated by FACScan laser flow cytometer (CyFlow^®^ Cube, Partec, Germany).

### Animal tMCAO model

Based on previously published methods, a transient middle cerebral artery occlusion (tMCAO) model was established in mice [35–38]. Forty male C57BL/6 mice (8–12 weeks old, 25–30 g) were obtained from the Experimental Animal Center of Capital Medical University (Beijing, China). Before surgery, mice were fasted 1 day before experiment for 5 h. Mice were anesthetized by 1.5% halothane in air using a face mask. In brief, the right common carotid artery, the external carotid artery, and the internal carotid artery were exposed. Then, middle cerebral artery occlusion (MCAO) was established by inserting a 2-cm-long nylon filament (diameter, 0.24–0.28 mm, Biospes, Chongqing, China) into a cut of the internal carotid artery. The body temperature of mice was maintained at 37 °C. To initiate blood flow reperfusion, the silicone-coated nylon filament was untied after 2 h of MCAO. Then, mice in the experimental group were treated with lncEPS-liposomes (50 µM/g, i.v.), lncEPS-leukosomes (50 µM/g, i.v.), or lincRNA-EPS (10 µg/g, i.v.). Mice in the sham group underwent the same procedure and treated with a similar volume of saline (i.v.). The number of animals per group was 10.

### ELISA

According to a previously reported method [39], the cerebro-spinal fluid (CSF) of mice was extracted at 1 week or 3 weeks after tMCAO. In addition, cell culture supernatant was collected after 2 days of culture. Then, levels of TNF-α, IL-6, and IL-1β in both cell culture supernatant and CSF were measured using ELISA kits (Bos-ter Bioengineering Co. Ltd. Wuhan, China). The minimum detection limits of the kits were 0.85 mg/ml for TNF-α, 13.7 pg/ml for IL-1β, and 22.9 pg/ml for IL-6. The results were obtained by a microplate reader (Synergy HT, BioTek, Winooski, VT, USA).

### Nitric oxide detection

The Griess reagent kit (Invitrogen) was used to measure total NO levels in the culture supernatant of each group [40]. Briefly, nitric oxide (NO) in the supernatant was reduced into nitrite, and nitrite reacted with the Griess reagent for 25 min at 37 °C in the dark. The NO levels were obtained by a microplate reader at a wavelength of 540 nm.

### Neuronal staining

At 1 week or 3 weeks after surgery, brains were removed, washed with refrigerated saline, and fixed for 12 h in 4% paraformaldehyde (Invitrogen). Next, brain tissue was transferred to 30% sucrose solution for 3 days. After dehydration, a series of brain sections (20 µm) was cut from the frontal to the occipital poles using a cryo-microtome (HM525, MICROM, Walldorf, Germany). Then, sections were stained with cresyl violet (176022, J&K Chemical, Beijing, China) as per a previously reported method [41].

In another series, brain tissue sections were incubated for 12 h at 4 °C with a mouse-anti-NeuN antibody (1:500, Chemicon, Temecula, CA, USA). After washing with PBS, sections were incubated with a secondary antibody, cy3-conjugated rabbit anti-mouse immunoglobulin G (IgG, 1:500, ThermoFisher, Waltham, MA, USA). The temporoparietal cortical layers III and IV were both located in the boundary zone and ischemic zone. Neurons were imaged using a fluorescence microscope at 525 nm.

### Immunofluorescence

To validate that the membrane proteins of Mφ were integrated within the liposome, leukosomes were incubated with the monoclonal antibody Rhoda-mouse-anti-CD86 (BD Biosciences) for 12 h at 4 °C. After high speed centrifugation (4 °C, 1000*g*, 1 h) and washing with PBS, leukosomes were imaged using a fluorescence microscope at 590 nm.

In addition, after fixation, brain sections from each group were incubated with the monoclonal antibody mouse-anti-Iba1 (BD Biosciences). Microglia were stained with a secondary FITC-conjugated rabbit anti-mouse IgG antibody for 90 min at room temperature. Microglia were stained using a mouse-anti-CD68 (BD Biosciences), followed by washing, and incubation with the secondary antibody Rhoda-mouse-IgG (ThermoFisher) as described above. Microglia were imaged using a fluorescence microscope at 525 nm and 590 nm.

### Statistical methods

Experimental values are presented as the mean ± standard deviation. One-way analysis of variance followed by the Student–Newman–Keuls post hoc test was used to determine statistically significant differences. Statistical evaluations were performed using GraphPad Prism statistical software (La Jolla, CA, USA). Data was considered statistically significant when *P *< 0.05.

## Results

### The incorporation of membrane proteins and lincRNA-EPS in lipid vesicles

**Fig. 1:**
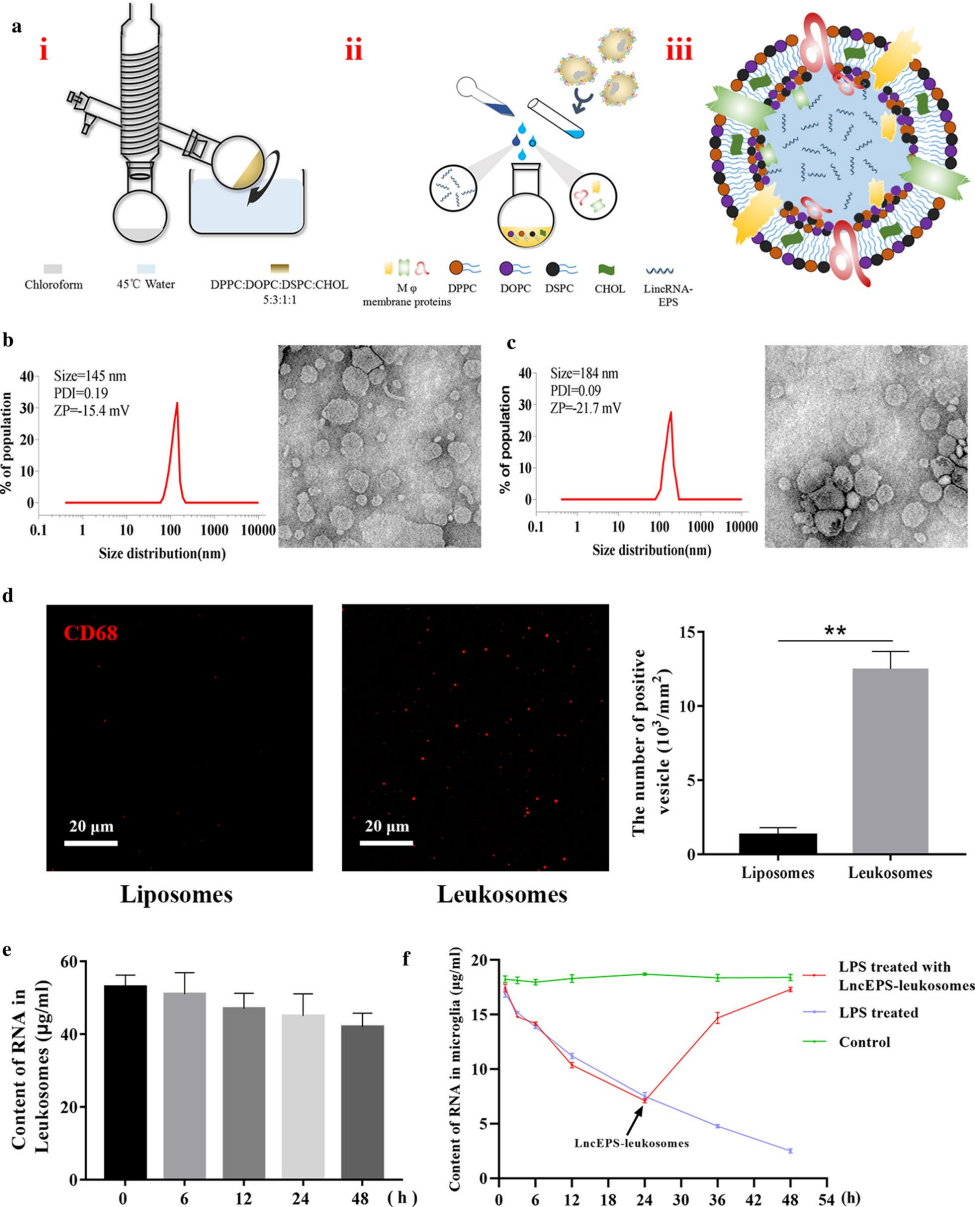
LncEPS-leukosome synthesis, formulation, and physicochemical features. **a** Liposome film was prepared by rotary vacuum evaporation. Extraction of membrane proteins from Mφ. Membrane proteins and lincRNA-EPS were loaded into the liposome. Pre-designed structure of LncEPS-leukosome. **b**, **c** The Dynamic Light Scattering (DLS) analysis show the size, zeta potential (ZP) and polydispersity index (PDI) of liposomes (**b**) and leukosomes (**c**). Cryo-TEM analysis show morphological of two formulations. **d** Immunofluorescence assay confirmed that membrane proteins were bound to the membrane of leukosomes. Liposomes and leukosomes were stained with an anti-CD86 antibody (red). **e** RT-PCR demonstrating the envelopment and leakage rate of lincEPS-leukosomes construed by 100 μg/ml lincRNA-EPS. **f** Changes in expression of microglial lncRNA-EPS after activation or treatment with lncEPS-leukosome were demonstrated by RT-PCR. Scale bar, 20 μm. (*n *= 8 per group). Values represent the mean ± SD, * and ^#^*P *< 0.05, ** and ^##^*P *< 0.01

lncEPS-leukosomes were constructed using phosphocholine-based phospholipids, cholesterol, membrane proteins of Mφ, and lincRNA-EPS. In our design for the structure of the vesicle, most proteins were combined with the liposome, and most of the lincRNA was packaged within liposomes (Fig. 1a).

After extrusion and dialysis, cryo-EM was used to determine shape the characteristics of the vesicles. The results demonstrated that most of the prepared liposomes and leukosomes were single-compartment liposomes, spherical in shape, with a uniform distribution. The detailed physical–chemical data showed that surface properties, size, and polydispersity index (PDI) were used by zeta potential and DLS. The zeta potential of two vesicles was − 15.7 mv and − 21.4 mv. The average size of liposomes was 145 mm, which was increased to 184 mm after mixed liposomes and membrane proteins were added. In addition, the PDI was 0.09 and 0.19 and leukosomes were bigger in size (P < 0.01) when compared to liposomes (Fig. 1b, c).

To evaluate whether membrane proteins of Mφ were combined with the envelope of leukosomes, leukosomes were evaluated by immunofluorescence using an antibody directed against CD68 (a specific marker for Mφ membrane proteins) under conditions that no treatment would rupture leukosomes. Liposomes and leukosomes were incubated with mouse-anti-CD68 antibody (BD Biosciences) at 4 °C for 12 h, washed with PBS and incubation with the secondary antibody Rhoda-mouse-IgG (ThermoFisher). The results showed that liposomes were not labeled. The numbers of liposomes and leukosomes were 0.07 × 10^3^/mm^2^ and 1.56 × 10^3^/mm^2^, respectively (Fig. 1d).

RT-PCR was used to measure the envelopment and leakage rate of lncEPS-leukosomes. The lincRNA-EPS content of lncEPS-leukosomes that were constructed with 100 μg/ml lincRNA-EPS was 52.3 μg/ml. This content at 6 h, 12 h, 24 h, and 48 h was 51.6 μg/ml, 50.2 μg/ml, 49.5 μg/ml, and 48.1 μg/ml respectively (Fig. 1e). In addition, the lincRNA-EPS content in microglia was measured. In the control group, the lincRNA-EPS content was maintained around 17.8 μg/ml. However, at 48 h after LPS treatment, the lincRNA-EPS content decreased to 3.45 μg/ml. Furthermore, at 24 h after LPS treatment, and when lncEPS-leukosomes were used to treat the microglia, the lincRNA-EPS content decreased to 7.38 μg/ml. After 48 h, the lincRNA-EPS content in microglia increased to 16.6 μg/ml (Fig. 1f).

### LincRNA-EPS is helpful for maintenance of the stemness of NSCs by inhibiting inflammation and abnormal migration of microglia

**Fig. 2:**
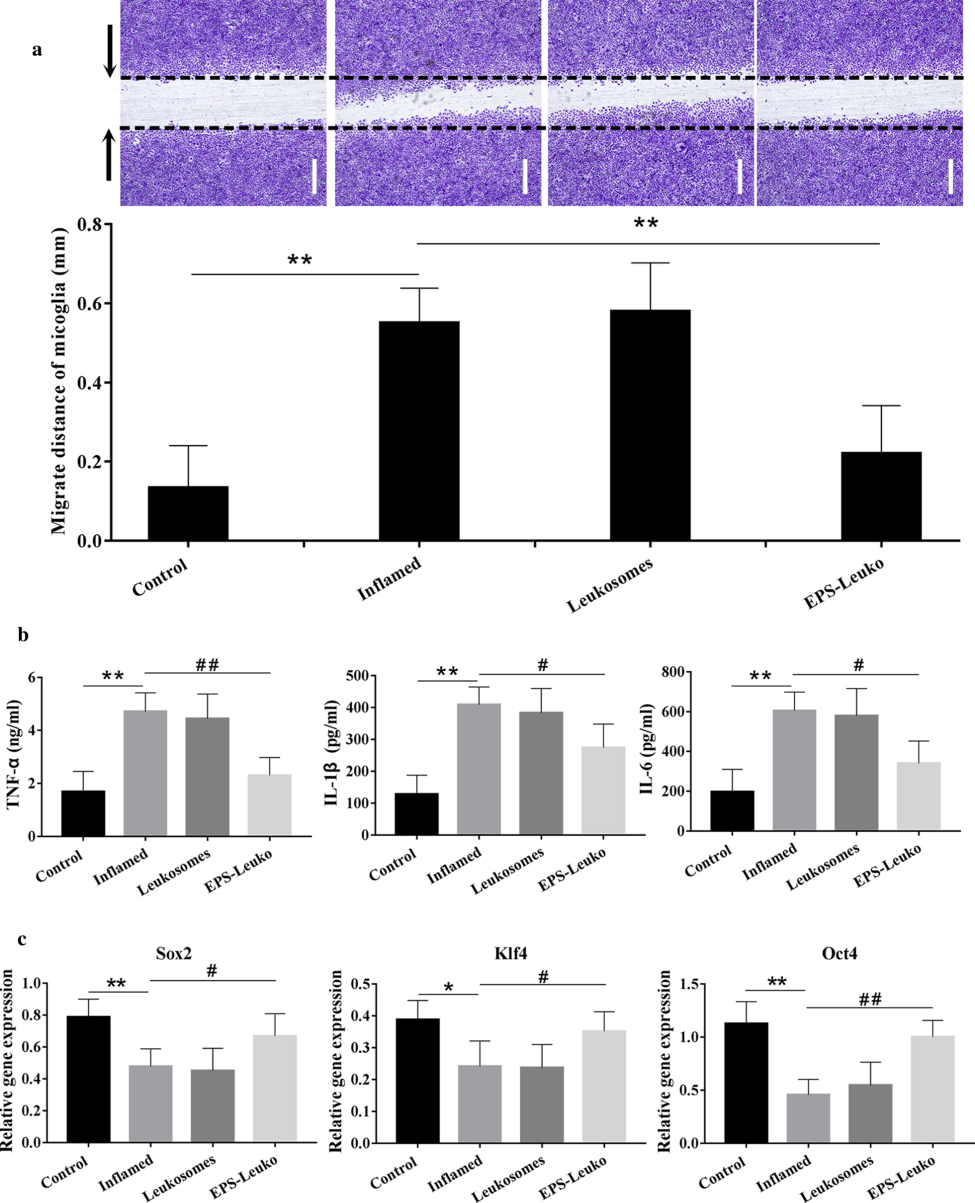
The effect of lincRNA-EPS on the over-activation of microglia and the maintenance of the stemness of NSCs. **a** A scratch wound was created and microglia were treated using lipopolysaccharide (LPS), leukosomes, or lncEPS-leukosomes. Representative images and quantification showed the migration of microglia. **b** LPS, leukosomes, or EPS-leuko (lncEPS-leukosomes) were added to the culture. ELISA was performed to determine the levels of TNF-α, IL-1β, and IL-6 in treated microglia. **c** Using the same treatment as mentioned above, gene expression of relative stemness (the expression of β-action is 1), including Klf4, Oct4, and Sox2 in NSCs were analyzed by RT-PCR (*n *= 8 per group). Values represent the mean ± SD, * and ^#^*P *< 0.05, ** and ^##^*P *< 0.01

The scratch test was used to determine the migration of microglia. Our results showed that the migration of microglia was low and the distance of migration at rest was only 0.173 mm. After treatment with LPS, the cell migration potential was strongly increased to 342.3%. Treatment with leukosomes did not significantly influence abnormal migration, and the distance of migration remained at 363.5%. Substituting leukosomes for lncEPS-leukosomes resulted in a significant decrease in the distance of migration to 142.9% (Fig. 2a).

The secreting levels of inflammatory factors that include TNF-α, IL-1β, and IL-6 were measured by ELISA. No obvious inflammatory reactions were identified, and the secreting levels of TNF-α, IL-1β, and IL-6 were 1.76 mg/ml, 128.2 pg/ml, and 203.7 pg/ml at rest. After treatment with LPS, the inflammatory reaction was significantly enhanced, and secreting levels were increased to 255.4%, 349.6%, and 278.5%, respectively. Moreover, treatment with leukosomes did not have a significant effect on the inhibition of inflammation and secreting levels of inflammatory factors remained similar with only slight changes. However, the secreting levels of TNF-α, IL-1β, and IL-6 strongly decreased to 146.4%, 227.5%, and 118.3% at 2 days after treatment with lncEPS-leukosomes (Fig. 2b).

In the control group, the relative gene expression of Sox2, Klf4, and Oct4 was 79.3%, 39.2%, and 113.7%, respectively. However, the results of RT-PCR showed that the gene expression of relative stemness that included Sox2, Klf4, and Oct4 in NSCs was decreased by LPS, and the expression levels decreased to 48.6%, 24.7% and 47.8%, respectively. After treatment with leukosomes, the gene expression level remained around 44.7%, 25.2% and 64.7%. However, lncEPS-leukosomes were helpful in maintaining the stemness of NSCs. The expression level of Sox2, Klf4, and Oct4 was increased to 69.1%, 37.3% and 98.5% after treatment with lncEPS-leukosomes (Fig. 2c).

### LincRNA-EPS is effective in reducing inflammation-induced apoptosis in NSCs, and promotes cell proliferation

**Fig. 3:**
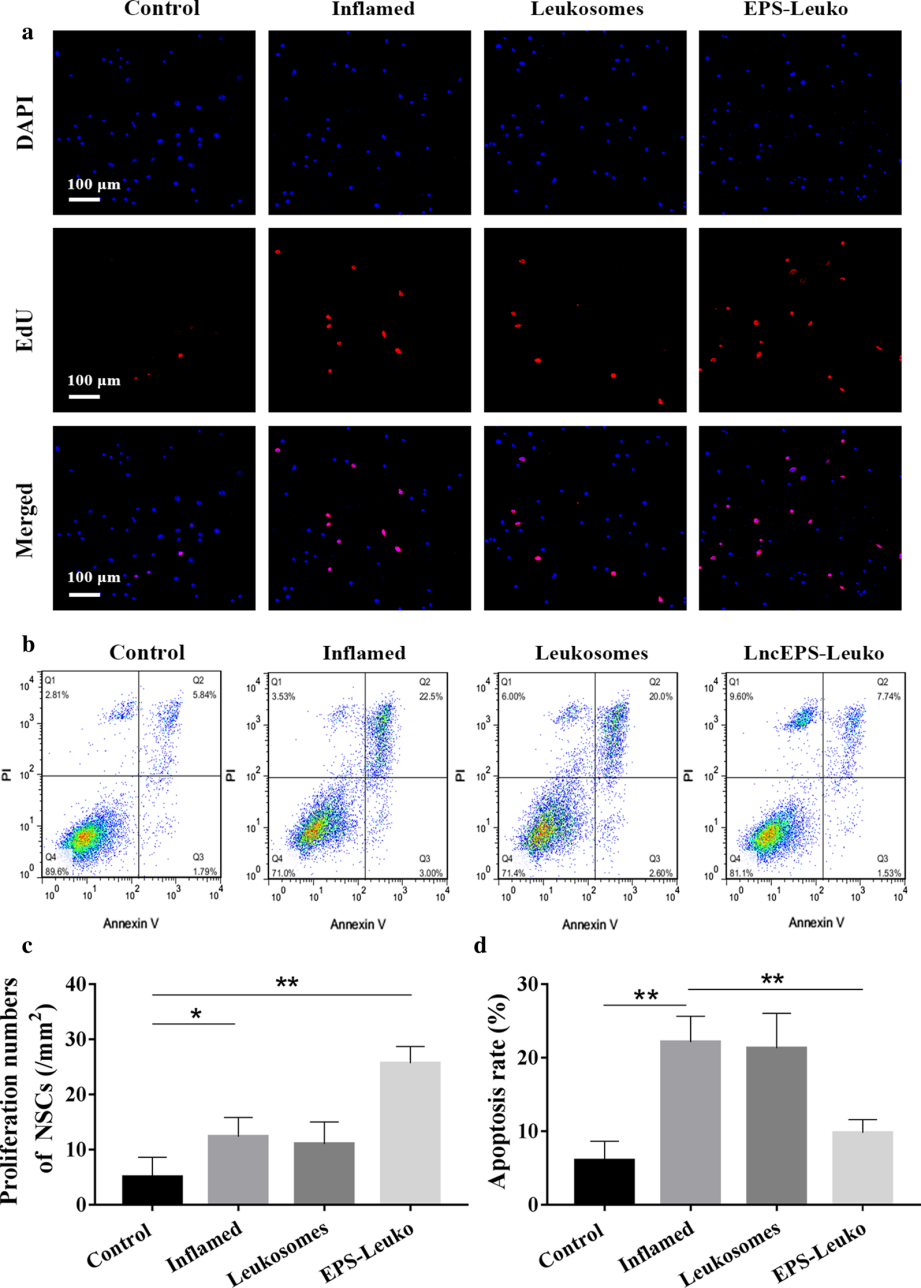
In the trans-well assay, microglia treated with lipopolysaccharide (LPS) were cultured in the upper chamber, and NSCs cultured in the lower chamber were treated with leukosomes or EPS-Leuko. **a** EdU cell proliferation assay showed the number of proliferating NSCs in different groups. **b** Annexin V-PI staining was used to determine apoptotic NSCs. The proportion of apoptotic cells was determined by flow cytometry. **c** Images of quantification showing the proliferation numbers of NSCs and apoptosis rate of NSCs in different groups. Scale bar, 100 μm (*n *= 8 per group). Values represent the mean ± SD, * and ^#^*P *< 0.05, ** and ^##^*P *< 0.01

Higher levels of inflammatory factors would promote proliferation of NSCs at early stages of ischemic-reperfusion, however long-lived inflammation increased cell apoptosis. In this study, changes in NSC proliferation were determined by an EdU cell proliferation bioassay. NSCs had a low proliferative potential, and the NSC number was 5.72/mm^2^ for sham treatment. In the inflamed group, the proliferative potential of NSCs was slightly improved, and the proliferation increased to 121.8%. After treatment with leukosomes, the proliferation of cells was 12.7/mm^2^, which was in line with that of the inflamed group. However, the proliferative potential was strongly improved and the proliferation increased to 554.4% after treatment with lncEPS-leukosomes (Fig. 3a, c).

In addition, apoptosis detection using Annexin V-PI was used to determine apoptotic NSCs. In the control group, the proportion of apoptotic cells was low (6.87%). In the inflamed group, however, the proportion of apoptotic cells was increased to 24.3%. The tendency of apoptotic cells was not improved after treatment with leukosomes, and the proportion of apoptotic cells remained 22.6%. However, the effect of inflammation on apoptosis of NSCs was inhibited by lncEPS-leukosomes, and the apoptotic proportion decreased to 11.2% (Fig. 3b, d).

### Leukosomes quickly transfer to the ischemic zone and infiltrate to microglia in tMCAO

**Fig. 4:**
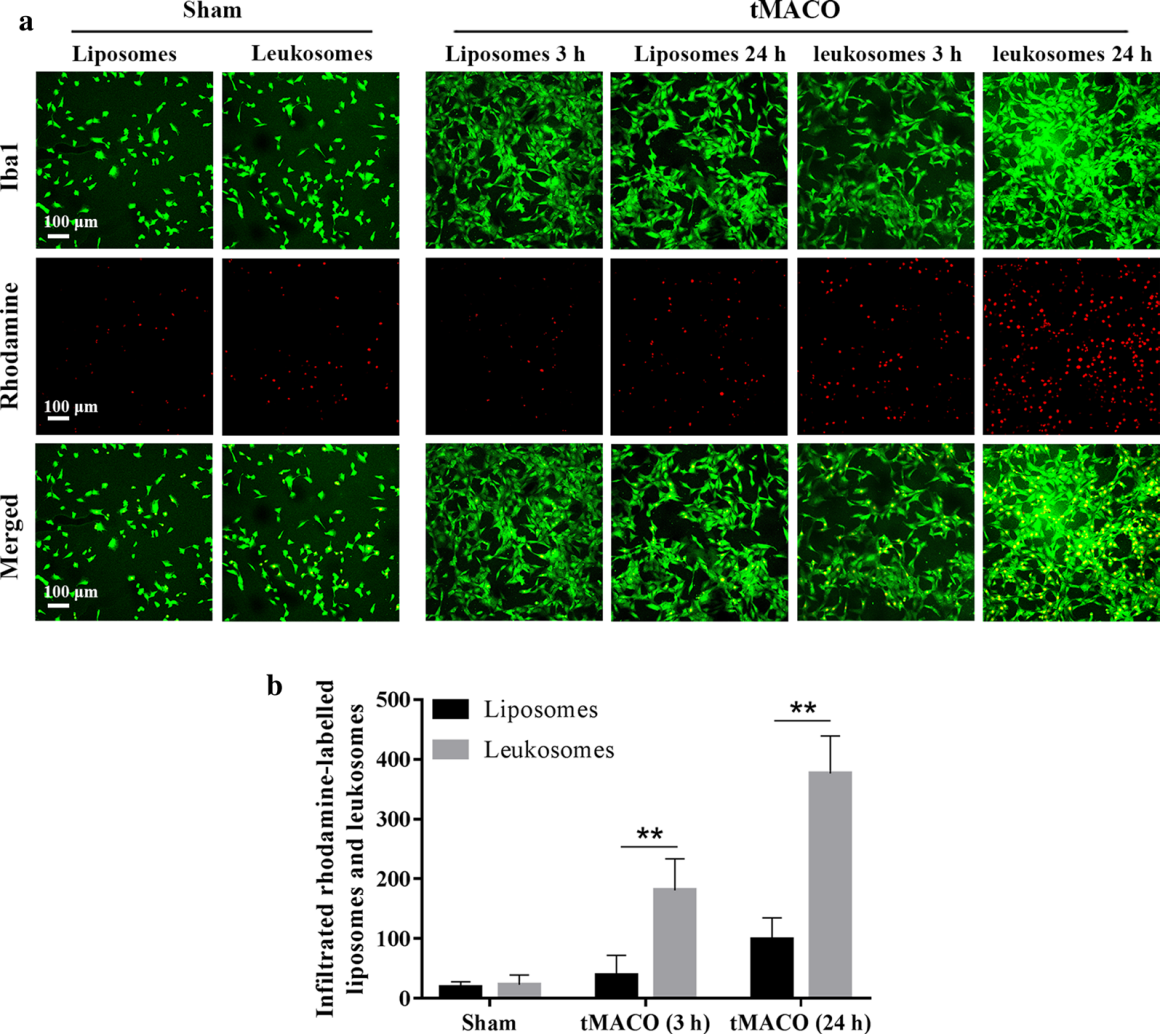
Ability of vesicles to metastasize and infiltrate. Mice were treated with liposomes or leukosomes at 2 h after tMCAO. Brain sections of mice were stained at 3 h and 24 h after tMCAO challenge. Prior to injection, vesicles were stained with rhodamine (red), whereas Iba1 (green) was used to stain microglia. **a**, **b** Representative images and quantification showing the transfer and infiltration of vesicles at 3 h and 24 h after tMCAO. Scale bar, 50 μm (*n *= 10 per group). Values represent the mean ± SD, * and ^#^*P *< 0.05, ** and ^##^*P *< 0.01

In this study, membrane proteins of Mφ were used as a targeted agent for the transfer of leukosomes. To measure the effect of membrane proteins on targeting, microglia in brain sections were stained with antibodies directed against Iba1, whereas liposomes and leukosomes were stained with Rhodamine. To verify the effect of lncEPS-leukosome in vivo, the method of thread bolt was used to establish a mouse model of tMCAO, in which mice were treated with lncEPS-liposomes (i.v.), lncEPS-leukosomes (i.v.), or lincRNA-EPS (i.v.) after 2 h of reperfusion. In the sham group, the ability of vesicles to metastasize was low and the total numbers of liposomes and leukosomes were 12.4/mm^2^ and 17.8/mm^2^, respectively. However, at 3 h after tMCAO, the ability of leukosomes to metastasize significantly improved, and the number of infiltrated vesicles increased to 738.6%. In addition, at 24 h after tMCAO, the ability of leukosomes to metastasize improved further and the number of vesicles increased to 1877.3%. At 3 h and 24 h after tMCAO, the ability of liposomes to metastasize slightly changed, and the number of liposomes represented 16.9% and 27.3% of the number of leukosomes. Our results showed that most leukosomes co-localized with microglia (Fig. 4a, b).

### LncEPS-leukosomes inhibit over-activation and infiltration of inflammatory cells in the tMCAO model

**Fig. 5:**
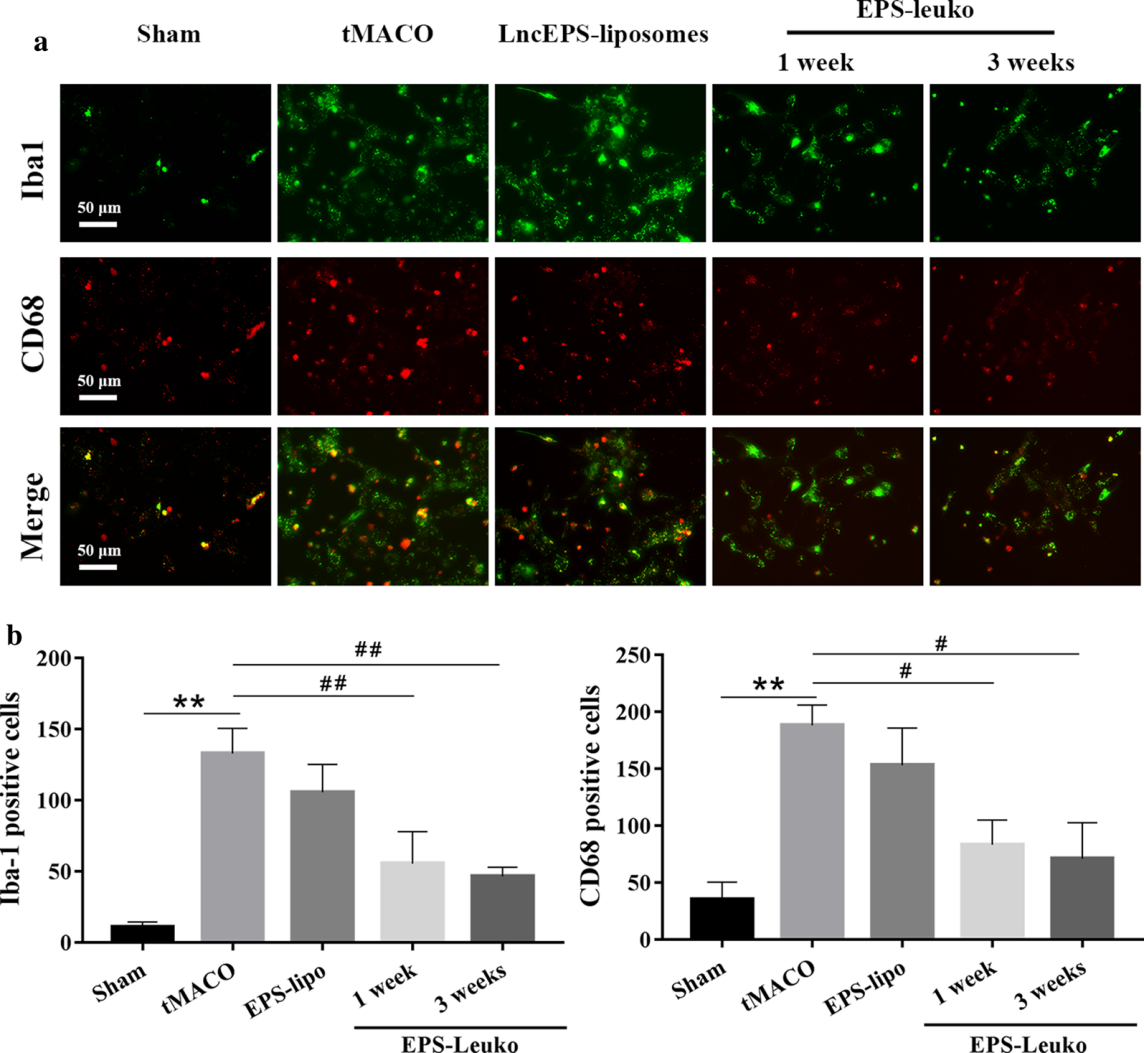
Activation and infiltration of inflammatory cells in the post-ischemic brain. Mice were treated with EPS-lipo (lncEPS-liposome) or EPS-Leuko at 2 h after tMCAO. In brain sections of the tMCAO model, Iba1 (red) was used to stain microglia, whereas CD68 (green) was used to stain with the homing macrophage. **a**, **b** Representative images and quantification showing activated inflammatory cells at 1 week and 3 weeks after tMCAO. Scale bar, 300 μm (*n *= 10 per group). Values represent the mean ± SD, * and ^#^*P *< 0.05, ** and ^##^*P *< 0.01

Cerebral ischemia and reperfusion results in over-activation and infiltration of inflammatory cells. To assess the effect of lncEPS-leukosomes on the infiltration of inflammatory cells, immunofluorescence assay was performed on brain sections using antibodies directed against Iba1 and CD68 to label inflammatory cells. Iba1 is a microglia specific marker, whereas CD68 is a homing macrophage-specific maker. In the sham group, the activation and infiltration levels of inflammatory cells were low, and the density of Iba1- and CD68-positve cells was 49.3/mm^2^ and 12.7/mm^2^, respectively. After tMCAO, both the activation of inflammatory cells as well as the infiltration levels were markedly increased, and the density of Iba1 and CD68-po strongly increased to 223.6% and 1782.9%, respectively. After treatment with lncEPS-liposomes for 3 weeks, there were several Iba1 and CD68 positive cells found in brain sections, which remained around 297.2% and 1684.6% in the sham group. However, after 1 week of treatment with lncEPS-leukosomes, the population of positive cells for Iba1 and CD68 strongly decreased to 157.4% and 543.5%. Activation of inflammatory cells and the level of infiltration were inhibited. After treatment with lncEPS-leukosomes for 3 weeks, the number of cells that were positive for Iba1 and CD68 decreased to 122.6% and 245.1%, and the positive cells were hardly detectable. Combined, these data showed that activation and infiltration of inflammatory cells was strictly inhibited and the level of inflammation in the brain was decreased (Fig. 5a, b).

### In the tMCAO model, lncEPS-leukosomes inhibit over-expression of inflammatory and cytotoxic factors

**Fig. 6:**
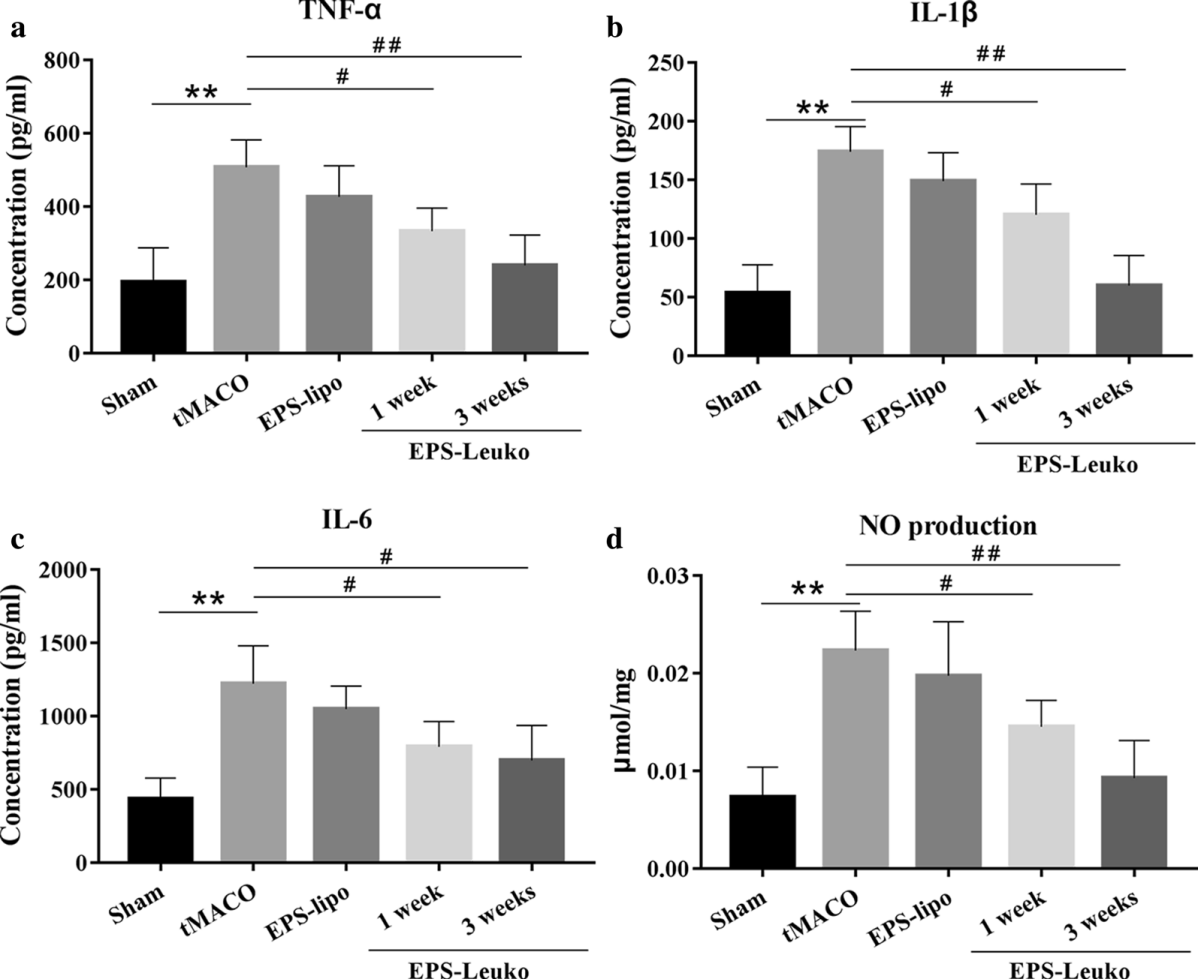
Expression levels of inflammatory factors in cerebro-spinal fluid. Mice were treated with EPS-lipo or EPS-Leuko at 2 h after tMCAO. Cerebro-spinal fluid (CSF) was extracted at 1 week and 3 weeks after tMCAO. ELISA assay and the Griess method were used to measure the expression levels of TNF-α, IL-6, IL-1β, and NO in CSF

Cerebral ischemia and reperfusion increased the number of inflammatory and cytotoxic factors expressed by inflammatory cells. To determine the effect of lncEPS-leukosomes on the expression of inflammatory and cytotoxic factors, the CSF was extracted and evaluated by ELISA. In the sham group, the expression of inflammatory and cytotoxic factors was performed in a functionally inactive situation, and the levels of TNF-α, IL-6, IL-1β, and NO were 198.6 pg/ml, 442.5 pg/ml, 53.9 pg/ml, and 0.0082 μmol/mg, respectively. After 3 weeks of tMCAO, we found a remarkable increase in the secretory activity of inflammatory cells and the levels of TNF-α, IL-6, IL-1β, and NO increased to 157.8%, 212.6%, 227.5%, and 185.1%, respectively. Treatment with lncEPS-liposomes had no effect on over-expression of inflammatory and cytotoxic factors. However, after 1 week of treatment with lncEPS-leukosomes, the expression of inflammatory and cytotoxic factors reduced, and the levels of TNF-α, IL-6, IL-1β, and NO decreased by 30.3%, 54.6%, 62.7%, and 57.5%, respectively. Furthermore, the expression of inflammatory and cytotoxic factors continued to be inhibited until 3 weeks after treatment, and levels was decreased by 43.6%, 31.5%, 90.4%, and 66.4%, respectively (Fig. 6).

### LncEPS-leukosomes promote neuron regeneration in the tMCAO model by inhibition of inflammation

**Fig. 7:**
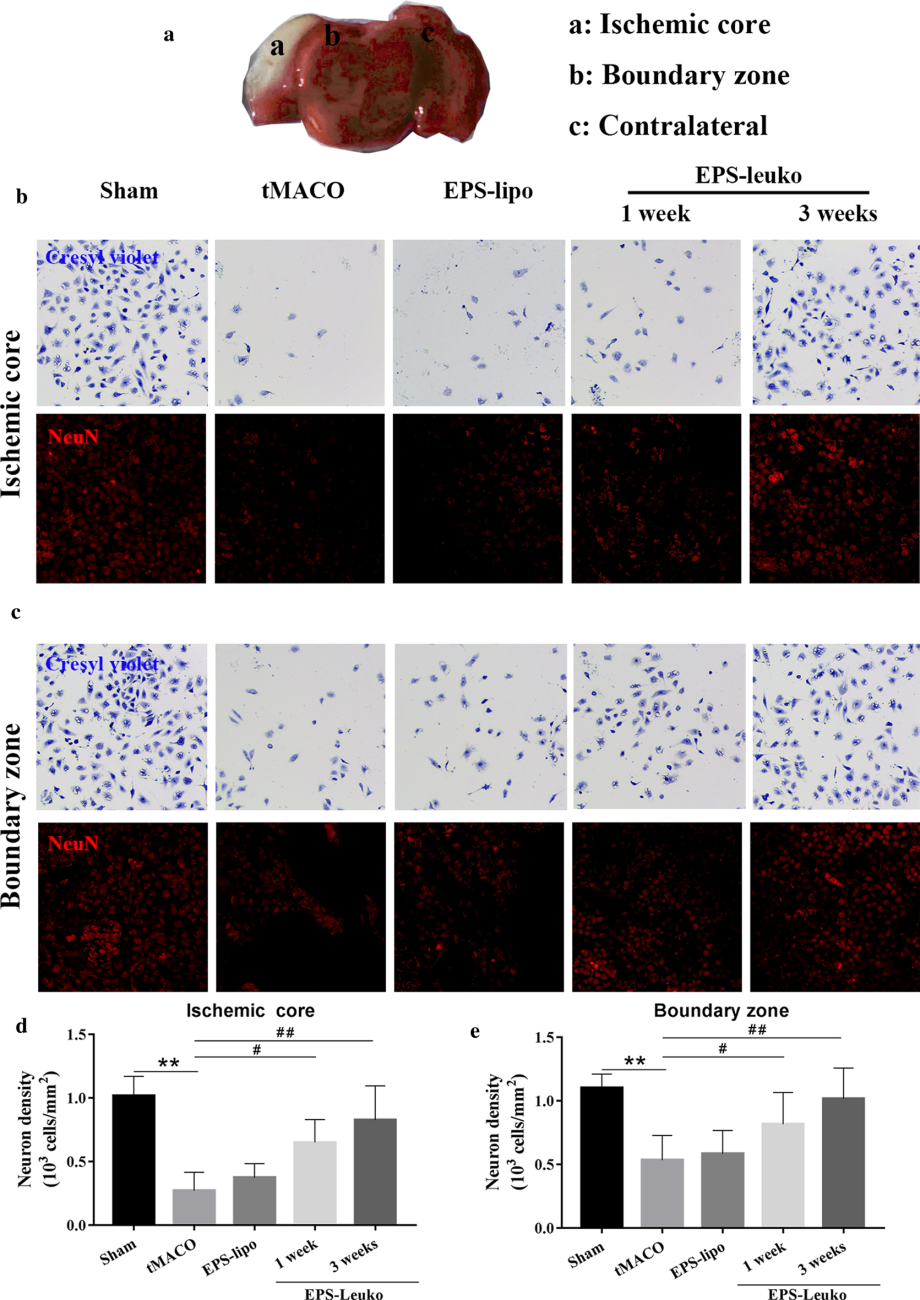
Effects of EPS-Leuko on neuronal density in the post-ischemic brain. Mice were treated as indicated at 2 h after tMCAO. The neuronal tissue of brain sections was stained with cresyl violet and NeuN (red) (Creyslviolet in upper rows, NeuN in bottom rows). Representative images and quantification imaging showed the neuronal density in the ischemic core (**a**, **c**) and boundary zone (**b**, **c**) at 1 week and 3 weeks after tMCAO. Scale bar, 50 μm (*n *= 10 per group). Values represent the mean ± SD, * and ^#^*P *< 0.05, ** and ^##^*P *< 0.01

Low levels of inflammation can result in neuronal damage but can also start the process of nerve repair and promote the proliferation of neural progenitor cells. Brain sections were processed with cresyl violet and NeuN immunofluorescence was performed to determine the proliferation of progenitor neurons. In the sham group, neurons located in the ischemic and boundary zone had a normal morphology and the cell density in both zones was 1.21 × 10^3^/mm^2^ and 1.22 × 10^3^/mm^2^, respectively. After 3 weeks of tMCAO, we found significant neuronal damage in the ischemic core and the boundary zone, and the cell density in both zones was decreased to 76.9% and 57.1%. After treatment with lncEPS-liposomes, no significant changes in neuronal density in ischemic and boundary areas were observed and in the tMCAO group, the cell density in both areas was maintained around 32.3% and 49.6%. However, after 1 week of treatment with lncEPS-leukosomes, neurons in the ischemic and boundary zone had a tendency of proliferation, and the neuronal density in both areas was increased to 0.73 × 10^3^/mm^2^ and 0.81 × 10^3^/mm^2^, respectively. Furthermore, the neuronal density in both areas were increased to 0.92 × 10^3^/mm^2^ and 1.18 × 10^3^/mm^2^. Together, the data showed that neuronal regeneration was observed in the ischemic hemisphere (Fig. 7).

## Discussion

Many studies have clearly shown that the main causes of neurological sequelae after stroke were over-activation of microglia and apoptosis of NSCs [42–44]. In this study, biomimetic vesicles were constructed by membrane proteins of Mφ, lincRNA-EPS, and liposomes. The effects of these vesicles on targeting inhibition of inflammation and promotion of neuronal regeneration were explored in vitro and in vivo.

Based on the well-accepted approach for the preparation of liposomes, we constructed biomimetic vesicle including Mφ membrane proteins and lincRNA-EPS, which represented an innovative structure and use of the targeted treatment for stroke. The results obtained by cryo-EM and DLS showed that leukosomes are bigger in size, the negativity of the zeta potential was decreased compared with liposomes, and the PDI was increased. Leukosomes had a more uniform particle size distribution and a better dispersion, which may be attributed to the combination and shielding effect of the proteins. Surface immunofluorescence assay showed that proteins can be successfully combined in the leukosome membrane. In addition, the lincRNA-EPS content of lncEPS-leukosomes did not change for 48 h, and the low content of lincRNA-EPS induced by treatment with LPS was significantly improved by lncEPS-leukosomes. These data suggested that vesicles can enhance the stability of lincRNA-EPS, and increase its delivery inside microglia.

In this study, the results obtained by the scratch test and ELISA showed that lncEPS-leukosomes could inhibit microglial abnormal migration and over secretion of inflammatory factors. On the other hand, the expression of Klf4, Oct4, and Sox2 was significantly increased after treatment with lncEPS-leukosomes. One explanation may be that inflammation subsides was helpful for maintaining the stemness of NSCs. Obviously, pure liposomes did not influence the inhibition of microglia activation and maintenance of the stemness of NSCs.

In vitro, an inflamed environment can promote NSC proliferation, however the proliferation was low and apoptosis was induced. We hypothesize that the inflammation may be a triggering factor for proliferation, and the inflammatory damage increases the level of apoptosis. After treatment with lncEPS-leukosomes, cell proliferation strongly increased and the apoptosis tendency was maintained, which may be explained by removing inflammatory factors. Our in vitro data confirmed that lncEPS was great for inflammatory inhibition and improving neuron regeneration.

The barrier effect of the BBB is the main problem of improving therapeutic effects [45, 46]. Moreover, drug targeting is the ideal solution to improve the therapeutic effects and reduce side effects. In the sham group and the tMCAO model, liposomes transferred to the ischemic zone because of their lipophilic surface characteristics. However, the number of vesicles was low. After treatment with lncEPS-leukosomes, we found that many vesicles transferred to the ischemic zone and most infiltrated to microglia. This showed that Mφ membrane proteins have a great targeting effect on inflammatory cells. We strongly believe that this targeting approach can be used for the treatment of other diseases.

After confirming the targeting effect of vesicles in the tMCAO model, we tested the full effect of lncEPS-leukosomes. The number of inflammatory cells, the level of inflammatory factors, and the level of cytotoxic factors were strongly decreased after treatment with lncEPS-leukosomes. In addition, the results showed that lncEPS-leukosomes inhibited over-activation and infiltration of inflammatory cells when compared with lncEPS-liposomes. The main cause is that the effect of lincRNA-EPS on inflammatory inhibition needs intracellular targeting. Moreover, our study showed why drug targeting is needed.

In the tMCAO model, we found that neurons in the ischemic and boundary zone showed an increase in death. It is suggested that diffuse inflammation can cause severe damage to the nervous system and that inflammation is not only limited to the ischemic zone. After treatment with lncEPS-leukosomes, neuronal regeneration continued for weeks. Although we have not tested the functionality of these neurons, the density of the neuron was near normal, thereby indicating sufficient neuron regeneration.

## Conclusions

In summary, we developed a novel targeting carrier for microglia with a lncEPS-loaded liposome. Through a series of experiments, we verified that targeting of Mφ membrane proteins for inflammatory cells and the effect of lncEPS-leukosomes on inflammation was induced by over-activated microglia. In addition, we demonstrated that the nervous system remained in a repair state for weeks with inflammation subsiding. Our lncEPS-leukosomes provide a novel approach to reduce the inflammatory reaction and accelerate neuron regeneration after cerebral apoplexy. The ideal concentration of lncEPS-leukosomes and the specific underlying mechanisms for the reduction of inflammation on nerve functional regeneration are unknown. This will be a major focus of our future studies.

## Data Availability

All data used to support the findings of this study have been presented in this manuscript.

## Abbreviations

LincRNA: Long intergenic noncoding RNA
tMCAO: Transient middle cerebral artery occlusion/reperfusion
NSC: Neural stem cell
BBB: Blood brain barrier
CSF: Cerebro-spinal fluid
